# Changes in the Prevalence and Correlates of Weight-Control Behaviors and Weight Perception in Adolescents in the UK, 1986-2015

**DOI:** 10.1001/jamapediatrics.2020.4746

**Published:** 2020-11-16

**Authors:** Francesca Solmi, Helen Sharpe, PhD, Suzanne H. Gage, Jane Maddock, Glyn Lewis, Praveetha Patalay

**Affiliations:** 1 Division of Psychiatry, University College London, London, United Kingdom; 2 School of Health in Social Science, University of Edinburgh, Edinburgh, United Kingdom; 3 Department of Psychological Sciences, University of Liverpool, Liverpool, United Kingdom; 4 MRC Unit for Lifelong Health and Ageing at University College London, Department of Population Science and Experimental Medicine, London, United Kingdom; 5 Centre for Longitudinal Studies and MRC Unit for Lifelong Health and Ageing, University College London, London, United Kingdom

## Abstract

**Importance:**

In the context of the growing prevalence of childhood obesity, behaviors aimed at weight loss and their psychological burden might be increasing.

**Objective:**

To investigate whether the prevalence of weight-control behaviors and weight perception, including their association with depressive symptoms, has changed in the 3 decades between 1986 and 2015.

**Design, Setting, and Participants:**

This study used data from repeated cross-sections from successive longitudinal birth cohort studies. These included general population samples of UK adolescents aged 14 to 16 years from 3 ongoing birth cohorts: the British Cohort Study 1970 (children born between April 5 and 11, 1970; data collected in 1986), the Avon Longitudinal Study of Parents and Children (mothers with expected delivery between April 1, 1991, and December 21, 1992; data collected in 2005), and the Millennium Cohort Study (children born between September 1, 2000, and January 11, 2002; data collected in 2015). A total of 22 503 adolescents with data available on at least 1 weight-control or weight-perception variable in midadolescence were included in the study. Data were analyzed from August 1, 2019, to January 15, 2020.

**Main Outcomes and Measures:**

Self-reported lifetime dieting and exercise for weight loss, current intentions about weight (doing nothing, lose weight, stay the same, gain weight), and weight perception (underweight, about the right weight, overweight) adjusted for body mass index. The secondary outcome was depressive symptoms.

**Exposures:**

The main exposure was time (ie, cohort); secondary exposures were weight-change behaviors and weight perception.

**Results:**

The study cohort included 22 503 adolescents (mean [SD] age, 14.8 [0.3] years; 12 061 girls [53.6%]; and 19 942 White individuals [89.9%]). A total of 5878 participants were from the British Cohort Study, 5832 were from the Avon Longitudinal Study of Parents and Children, and 10 793 were from the Millennium Cohort Study. In 2015, 4809 participants (44.4%) had dieted and 6514 (60.5%) had exercised to lose weight compared with 1952 (37.7%) and 344 (6.8%) in 1986. Furthermore, 4539 (42.2%) were trying to lose weight in 2015 compared with 1767 (28.6%) in 2005. Although girls were more likely to report these behaviors in all years, their prevalence increased more in boys over time (lifetime dieting in boys: odds ratio [OR], 1.79; 95% CI, 1.24-2.59; in girls: OR, 1.23; 95% CI, 0.91-1.66; currently trying to lose weight in boys: OR, 2.75; 95% CI, 2.38-3.19; in girls: OR, 1.70; 95% CI, 1.50-1.92). Adolescents also became more likely to overestimate their weight (boys describing themselves as overweight adjusting for body mass index, 2005 vs 1985 OR, 1.60; 95% CI, 1.17-2.19; 2015 vs 1985 OR, 1.36; 95% CI, 1.04-1.80; girls describing themselves as underweight, after adjusting for body mass index, 2015 vs 1986 OR, 0.51; 95% CI, 0.28-0.91). Girls who described themselves as overweight experienced increasingly greater depressive symptoms over time compared to girls who described their weight as about right (mean difference 1986, 0.32; 95% CI, 0.22-0.41; mean difference 2005, 0.33; 95% CI, 0.24-0.42; mean difference 2015, 0.56; 95% CI, 0.49-0.62).

**Conclusions and Relevance:**

These findings suggest that the growing focus on obesity prevention might have had unintended consequences related to weight-control behaviors and poor mental health. Public health campaigns addressing obesity should include prevention of disordered eating behaviors and be sensitive to negative impact on mental health.

## Key Points

**Question:**

Has the prevalence of behaviors aimed at weight loss and weight perception in adolescents and their association with depressive symptoms changed over 30 years?

**Findings:**

This study, which used data from a total of 22 503 adolescents in 3 UK cohorts spanning 30 years, found that the prevalence of behaviors aimed at achieving weight loss increased in 2015 compared with both 2005 and 1986 and that this change was not explained by known changes in body mass index alone. Weight-control behaviors increased more in boys than in girls, but among the latter, these behaviors were associated with greater depressive symptoms in 2015 compared with 2005 and 1986.

**Meaning:**

These findings suggest that an increased societal and public health focus on obesity could have had unintended consequences related to weight-control behaviors and poor mental health; thus, public health campaigns around the prevention of obesity should include prevention of disordered eating behaviors and be sensitive to adverse outcomes such as poor mental health.

## Teaser

This cohort study uses data from 3 cohort studies in the UK to investigate the changes in prevalence of adolescent weight-loss and weight-perception behaviors and their association with depressive symptoms.

## Introduction

The proportion of adolescents with an overweight or obese body mass index (BMI) has almost tripled over the past 40 years in the UK.^1,2^ Approximately 40% of UK adolescents aged 13 to 15 years have an overweight or obese BMI.^2^ Government strategies for the prevention of obesity in childhood include raising awareness of food caloric intake (eg, the “traffic light” system on food packaging),^3^ introducing the Soft Drinks Industry Levy in 2018,^3^ and increasing physical activity.^4^

Restrictive eating behaviors aimed at weight loss can be common in adolescence, particularly among adolescents who have an overweight BMI.^5^ Because of the increasing prevalence of obesity and widespread societal messages promoting thinness, restrictive eating behaviors may be becoming more common across the BMI spectrum. This is of concern because experimental studies have found that dieting is ineffective at reducing body weight in young people^6^ and that restrictive eating behaviors are longitudinally associated with adverse mental health outcomes, including depression and eating disorders.^7,8,9,10,11,12,13^

We are not aware of any UK general population studies investigating time trends in weight-control behaviors and weight perception in adolescence and changes to their psychological correlates. Recently, the UK government has highlighted these issues as an area of increasing policy concern.^14^ Findings from other Western countries^15,16,17,18^ provide inconsistent evidence. Data from the US, Norway, Cyprus, Sweden, and New Zealand show an increase in the proportion of weight-control behaviors in early adolescence, particularly in boys.^15,16^ However, 2 studies based in the US and Finland found that the prevalence of weight-control behaviors did not change over a 10-year time period (US, 1999-2010; Finland, 2003-2013),^19,20^ although girls in Finland became more likely to believe they would feel worthless if they could not achieve their desired weight.^19^ This suggests that the psychological burden associated with these behaviors might have increased over time.

In this study, we used harmonized data spanning 30 years derived from 3 UK birth cohorts that collected data in midadolescence on weight-perception and weight-control behaviors in 1986, 2005, and 2015, with 2 aims. First, we examine whether the prevalence of weight-control behaviors and weight perception changed in the 3 decades between 1986 and 2015 and whether any changes vary by sex. Second, we estimate their related psychological burden by investigating their associations with depressive symptoms and whether the magnitude of these associations has changed over time.

## Methods

### Participants

We used data from 3 ongoing UK cohorts: the 1970 British Cohort Study (BCS; children born between April 5 and 11, 1970), the Avon Longitudinal Study of Parents and Children (ALSPAC; mothers with expected delivery between April 1, 1991, and December 31, 1992), and the Millennium Cohort Study (MCS; children born between September 1, 2000, and January 11, 2002; details in eMethods 1 in the Supplement) collected when participants were aged approximately 16 years (BCS, in 1986) or 14 years (ALSPAC in 2005 and MCS in 2015). Henceforth, we refer to each cohort by the year at which the outcomes were measured (1986, 2005, 2015). Ethics approval for BCS was obtained for all sweeps after the year 2000. Prior sweeps received internal approval in line with the regulations of the time.^21^ The ALSPAC Law and Ethics committee, the Local Research Ethics committees, and the Multi-Centre Research Ethics Committee gave ethical approval for ALSPAC and MCS. Participants gave written consent to take part in the studies.

In our sample, we included participants with data available on at least 1 of the main weight-change or weight-perception outcomes. In the case of twins (BCS: n = 199; ALSPAC: n = 202; MCS: n = 246 twins and n = 10 triplets), we retained 1 participant per twin or triplet at random, as their shared genetic and environmental exposures might otherwise lead to over- or underestimation of the associations. As not all outcomes were measured in all cohorts (eTable 1 in the Supplement), our analytical samples vary depending on the analyses of interest. This study followed the Strengthening the Reporting of Observational Studies in Epidemiology (STROBE) reporting guideline.

### Weight-Control Behaviors and Weight Perception

The ALSPAC and MCS surveys asked what the adolescent was trying to do about their weight (not doing anything, stay the same, lose weight, or gain weight). We harmonized questions on lifetime exercising for weight loss and dieting (responses yes or no) in BCS and MCS (eTable 1 in the Supplement). We also used a harmonized variable (in ALSPAC, MCS, and BCS) indicating whether adolescents perceived themselves as underweight, about the right weight, or overweight (eTable 1 in the Supplement).

### Depressive Symptoms

In ALSPAC and MCS, depressive symptoms were assessed using the 12-item Short Moods and Feelings Questionnaire (SMFQ)^22^ and in BCS using the 9-item Malaise Inventory^23^ (eMethods 2 in the Supplement). We used cohort-standardized scores from these scales as a measure of the sample distribution of depressive symptoms in each cohort.

### Other Variables

Given geographical and sociodemographic differences between cohorts,^24^ we controlled our analyses for key sociodemographic characteristics, including age, sex, and racial/ethnic group (White British or European/ethnic minority), maternal age at birth, maternal education (compulsory vs noncompulsory), and paternal (where missing maternal) social class (manual vs nonmanual profession). Some of our analyses were adjusted for age- and sex-standardized BMI (eMethods 3 in the Supplement).^25,26,27^

### Statistical Analysis

We imputed missing data for participants with at least 1 outcome variable available using multiple imputation by chained equations, imputing 50 data sets.^28^ We additionally created attrition weights as the inverse of the probability of having taken part in the sweep of interest to account for attrition from baseline. We ran all analyses in imputed data sets using attrition weights (eMethods 4 in the Supplement). Given aggregated cross-cohort analyses, we could not account for the stratified sampling of the MCS. As a sensitivity check, we calculated overall and sex-stratified prevalence of weight-related behaviors and regressions accounting for stratified sampling for this cohort (and found identical results).

To investigate the presence of cohort effects in outcome prevalence, we used multinomial logistic or logistic regression models (depending on the outcome variable) including a categorical exposure indicating to which cohort the participant belonged (BCS, ALSPAC, or MCS). To account for sociodemographic differences between cohorts, we then ran a multivariable model adjusting for child’s sex, age, and race/ethnicity; maternal age and education; and paternal social class. We also tested for sex differences with interaction terms between sex and cohort.

We adjusted analyses of weight perception for BMI as we were interested in exploring changes in perception given known cohort differences in BMI.^24^ Controlling for BMI can indicate whether the prevalence of attempts at weight loss or gain has changed independently of BMI. We also adjusted analyses of current behaviors (“What are you trying to do about weight?”) for BMI in a separate mode l (eTable 5 in the Supplement). We did not adjust analyses of lifetime dieting and exercising for BMI because we could not know whether BMI at assessment was a risk factor for or a consequence of these behaviors. Our second aim was to investigate whether the magnitude of the cross-sectional associations of weight-change behaviors and weight perception with depressive symptoms had changed over time. We first ran univariable and multivariable linear regression models, adjusting for all covariates plus BMI. We then investigated the presence of sex- and cohort-specific changes over time with interaction terms between sex and cohort and weight-related behaviors. In all analyses, if there was evidence of an interaction, we presented all subsequent analyses stratified by sex. We performed all analyses from August 1, 2019, to January 15, 2020, using Stata software, version 15 (StataCorp LLC).

We preregistered the analytical plan of the study on the Open Science Framework on July 25, 2019.^29^ We report minor deviations from this in eMethods 5 in the Supplement.

## Results

In total, 22 503 adolescents (5878 from BCS, 5832 from ALSPAC, and 10 793 from MCS; mean [SD] age, 14.8 [0.3] years; 12 061 [53.6%] girls; and 19 942 White individuals [89.9%]) had at least 1 outcome variable available and were included in our study (eTable 2 in the Supplement). By the midadolescence assessment, loss to follow-up was 41.8% in MCS (n = 7756), 57.7% in ALSPAC (n = 7956), and 65.4% in BCS (n = 11 099). Factors associated with loss to follow-up are listed in eTable 3 in the Supplement.

### Lifetime Dieting and Exercising for Weight Loss

In 1986, 1952 adolescents (37.7%) reported having dieted and 344 (6.8%) exercised for weight loss, compared with 4809 (44.4%) and 6514 (60.5%) in 2015 (eTable 4 in the Supplement). At both times, a higher percentage of girls than boys reported these behaviors (dieting in 1986 among boys: 17.5% [95% CI, 15.9%-19.2%]; among girls: 59.2% [95% CI, 57.3%-61.1%] vs dieting in 2015 among boys: 34.6% [95% CI, 33.2%-35.9%]; among girls, 55.1% [95% CI, 53.8%-56.4%]; exercising in 1986 among boys: 4.9% [95% CI, 3.9%-5.9%]; among girls, 8.8% [95% CI, 7.7%-10.0%] vs exercising in 2015 among boys: 54.9% [95% CI, 53.6%-56.3%]; among girls, 66.3% [95% CI, 65.0%-67.6%]) (Table 1). There was an overall increase in dieting in 2015 compared with 1986, which differed by sex; boys showed a larger increase in dieting (odds ratio [OR], 1.79; 95% CI, 1.24-2.59) than girls (OR, 1.23; 95% CI, 0.91-1.66) (Table 2). There was also evidence of a large increase in the prevalence of exercising to lose weight in 2015 compared with 1986, which did not vary by sex (OR, 26.67; 95% CI, 20.06-35.40).

**Table 1.** Prevalence of Dieting, Exercising for Weight Loss, and Intention to Lose or Gain Weight by Participant’s Sex and Cohort (Based on Imputed Data Set With Attrition Weights)

Variable	% (95% CI)					
	1986	2005	2015	1986	2005	2015
Lifetime dieting	Boys (n = 7850)			Girls (n = 8835)		
No	82.5 (80.8-84.1)	NA	65.4 (64.1-66.7)	40.8 (38.9-42.7)	NA	44.9 (43.6-46.2)
Yes	17.5 (15.9-19.2)	NA	34.6 (33.2-35.9)	59.2 (57.3-61.1)	NA	55.1 (53.8-56.4)
Lifetime exercising for weight loss	Males (n = 7850)			Females (n = 8835)		
No	95.1 (94.1-96.1)	NA	45.1 (43.7-46.4)	91.2 (90.0-92.3)	NA	33.7 (32.4-35.0)
Yes	4.9 (3.9-5.9)	NA	54.9 (53.6-56.3)	8.8 (7.7-10.0)	NA	66.3 (65.0- 67.6)
What are you trying to do about weight?	Boys (n = 7930)			Girls (n = 8698)		
Nothing	NA	47.0 (45.0-49.0)	27.1 (25.9-28.3)	NA	28.7 (27.1-30.3)	20.4 (19.3-21.5)
Lose weight	NA	19.4 (17.8-21.0)	31.8 (30.6- 33.1)	NA	40.3 (38.6-42.1)	52.8 (51.1-54.5)
Stay the same	NA	26.2 (24.5-28.0)	28.3 (27.1-29.6)	NA	28.0 (26.4-29.6)	22.5 (21.1-23.9)
Gain weight	NA	7.3 (6.3-8.3)	12.7 (11.8- 13.6)	NA	2.9 (2.3-3.6)	4.1 (3.6-4.6)

Abbreviation: NA, not applicable.

**Table 2.** Univariable and Multivariable Logistic Regression Models Testing Cohort Effects in the Prevalence of Lifetime Dieting and Exercising for Weight Loss and Interactions With Adolescent’s Sex

Variable	OR (95% CI)		Cohort × sex interaction, *P* value	Multivariable model, OR (95%CI)	
	Univariable model	Multivariable^a^ model		Boys	Girls
Lifetime dieting for weight loss^b^ (comparing 2015 to 1986)					
Yes (2015 vs 1986)	1.33 (1.24-1.43)	1.55 (1.23-1.95)	<.001	1.79 (1.24-2.59)	1.23 (0.91-1.66)
Lifetime exercising for weight loss^b^ (comparing 2015 to 1986)					
Yes (2015 vs 1986)	20.92 (18.42-23.75)	26.67 (20.06-35.40)	.27	NA	NA
What are you currently trying to do about your weight? (comparing 2015 to 2005, RRR [95% CI])^c^					
Doing nothing	1 [Reference]	1 [Reference]	NA	1 [Reference]	1 [Reference]
Lose weight (2015 vs 2005)	2.29 (2.10-2.48)	2.18 (1.98-2.38)	<.001	2.75 (2.38-3.19)	1.70 (1.50-1.92)
Stay same (2015 vs 2005)	1.51 (1.38-1.64)	1.52 (1.38-1.68)	<.001	1.89 (1.63-2.16)	1.15 (1.00-1.32)
Gain weight (2015 vs 2005)	2.62 (2.24-3.05)	1.99 (1.67-2.36)	.01	2.32 (1.89-2.85)	1.53 (1.14-2.07)
Do you think you are: (comparing 2015 and 2005 to 1986, RRR [95% CI])^d^					
Underweight (2005 vs 1986)	1.29 (1.15-1.46)	1.49 (1.00-2.21)	.73	1.89 (1.12-3.18)	1.00 (0.54-1.82)
Underweight (2015 vs 1986)	0.64 (0.57-0.72)	0.72 (0.51-1.03)	.22	0.97 (0.61-1.53)	0.43 (0.25-0.76)
About the right weight	1 [Reference]	1 [Reference]	NA	1 [Reference]	1 [Reference]
Overweight (2005 vs 1986)	1.36 (1.24-1.49)	1.64 (1.22-2.19)	<.001	3.07 (1.82-5.15)	1.01 (0.71-1.44)
Overweight (2015 vs 1986)	1.66 (1.54-1.79)	1.47 (1.14-1.89)	<.001	2.59 (1.66-4.06)	0.95 (0.69-1.30)

Abbreviations: NA, not applicable; OR, odds ratio; RRR, relative risk ratio.

^a^ Adjusted for adolescent’s sex and ethnicity, maternal age and highest level of education, and paternal social class. Analyses of the question “Do you think you are?” are additionally adjusted for BMI.

^b^ Sample size, n = 16 671.

^c^ Sample size, n = 16 625.

^d^ Sample size, n = 22 503.

### Weight-Loss and Weight-Gain Attempts

In 2015, a greater proportion of adolescents said that they were trying to lose (4539 [42.2%]) or gain (894 [8.5%]) weight compared with 2005 (1767 [29.8%] and 286 [5.2%]) (eTable 4 in the Supplement). At both times, more girls said they were trying to lose weight compared with boys (lose weight: 2015 girls, 52.8% [95% CI, 51.1%-54.5%] vs boys, 31.8% [95% CI, 30.6%-33.1%]; lose weight: 2005 girls, 40.3% [95% CI, 38.6%-42.1%] vs boys, 19.4% [95% CI, 17.8%-21.0%]), whereas more boys than girls said that they were trying to gain weight (gain weight: 2015 girls, 4.1% [95% CI, 3.6%-4.6%] vs boys, 12.7% [95% CI, 11.8%-13.6%]; gain weight: 2005 girls, 2.9% [95% CI, 2.3%-3.6%] vs boys, 7.3% [95% CI, 6.3%-8.3%]) (Table 1). In regression analyses accounting for confounders, compared with 2005, in 2015, adolescents were more likely to say that they were trying to lose weight, gain weight, or stay the same weight than to say that they were doing nothing about their weight; these differences were greater for boys than girls across all 3 outcomes (lose weight: OR, 2.75 [95% CI, 2.38-3.19] for boys vs OR, 1.70 [95% CI, 1.50-1.92] for girls; stay the same: OR, 1.89 [95% CI, 1.63-2.16] for boys vs OR, 1.15 [95% CI, 1.00-1.32] for girls; and gain weight: OR, 2.32 [95% CI, 1.89-2.85] for boys vs OR, 1.53 [95% CI, 1.14-2.07] for girls) (Table 2). When we additionally adjusted for BMI, results did not change substantially (eTable 5 in the Supplement).

### Weight Perception

In 2005 (n = 1570 [27.0%]) and 2015 (n = 3571 [33.4%]), a greater proportion of adolescents said that they thought they were overweight than in 1986 (n = 1298 [22.2%]) (eTable 4 in the Supplement). Compared with 1986 and 2005, in 2015, more girls with underweight BMI thought their weight was “about right” (2015: 60.8% [95% CI, 55.4%-66.3%]; 2005: 42.3% [95% CI, 36.0%-48.6%]; 1986: 51.7% [95% CI, 46.2%-57.1%]) whereas more boys—both in 2005 and 2015—in the normal BMI range thought they were overweight compared with 1986 (2015: 11.5% [95% CI, 10.5%-12.6%]; 2005: 11.5% [95% CI, 10.0%-13.0%]; 1986: 7.3% [95% CI, 6.1%-8.5%]) (eTable 6 in the Supplement). Similarly, the proportion of boys, but not girls, with overweight BMI who thought they were overweight increased in 2005 (74.5% [95% CI, 70.2%-78.8%]) compared with 1986 (60.3% [95% CI, 54.1%-66.6%]) and remained stable in 2015 (73.1% [95% CI, 70.7%-75.5%]). After adjusting for BMI and other confounders, compared with 1986, adolescents were more likely to think they were overweight and less likely to think they were underweight in 2015 (overweight: OR, 1.47 [95% CI, 1.14-1.89] vs underweight: OR, 0.72 [95% CI, 0.51-1.03]) (Table 2). At any level of BMI, boys, but not girls, were more likely to think they were overweight (vs the right weight) in 2005 (boys: OR, 3.07 [95% CI, 1.82-5.15] vs girls: OR, 1.01 [95% CI, 0.71-1.44]) and 2015 (boys: OR, 2.59 [95% CI, 1.66-4.06] vs girls: OR, 0.95 [95% CI, 0.69-1.30]) compared with 1986. Boys were also more likely to think they were underweight (vs the right weight) in 2005 compared with 1986 (OR, 1.89 [95% CI, 1.12-3.18]) but no longer in 2015 (OR, 0.97 [95% CI, 0.61-1.53]). In contrast, girls in 2015 became less likely to think they were underweight (OR, 0.43 [95% CI, 0.25-0.76]) compared with 1986, meaning that even at low BMI values, they thought they were “about the right weight.” However, we did not find a similar association when comparing 2005 with 1986 (OR, 1.00 [95% CI, 0.54-1.82]).

### Associations of Weight-Change Behaviors and Weight Perception With Depressive Symptoms

**Figure 1.**
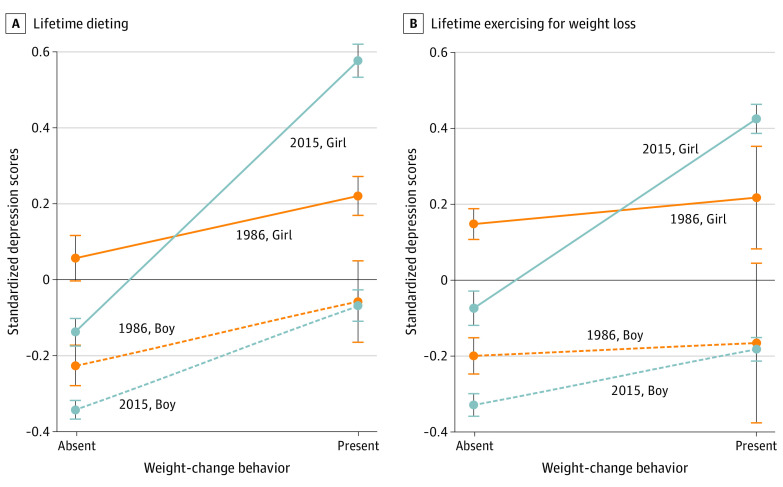
Changes in the Association Between Weight-Change Behaviors and Depressive Symptoms by Cohort and Sex The figure shows how the association of lifetime dieting (A) and lifetime exercising (B) for weight loss with depressive symptoms changed across cohorts in boys and girls. Parameters are derived from multivariate linear regression models in Table 3 with exposure × cohort interaction and sex-stratified analyses presented in eTable 7 in the Supplement.

Adolescents who dieted or exercised for weight loss and those who were trying to lose weight had greater depressive symptoms (dieting mean difference: 0.37 [95% CI, 0.33-0.41]; exercise mean difference: 0.26 [95% CI, 0.22-0.30]) (Table 3). The magnitude of these associations differed by sex, with girls engaging in these behaviors reporting greater levels of depression than boys (dieting mean difference girls: 0.49 [95% CI, 0.43-0.54]; boys: 0.26 [95% CI, 0.20-0.32]; exercise mean difference girls: 0.38 [95% CI, 0.32-0.44]; boys: 0.13 [95% CI, 0.08-0.19]). There was also evidence that girls who had dieted or exercised for weight loss in 2015 had greater depressive symptoms than those who engaged in these behaviors in 1986 (mean difference dieting 2015: 0.72 [95% CI, 0.66-0.88] vs 1986: 0.21 [95% CI, 0.12-0.29]; exercising 2015: 0.46 [95% CI: 0.39-0.53] vs 1986: 0.06 [95% CI, –0.08 to 0.20]), with weaker evidence of difference observed for those who were currently trying to lose weight in 2015 compared with 2005 (mean difference 2015: 0.58 [95% CI, 0.50-0.66] vs 2005: 0.43 [95% CI, 0.31-0.54]). There was no evidence of such differences in boys (Figure 1; eTable 7 in the Supplement).

**Table 3.** Univariable and Multivariable Linear Regression Models Testing the Association Between Dieting, Exercising for Weight Loss, Weight Intentions and Weight Perception With Depressive Symptoms^a^

Depressive symptom	Mean difference (95% CI)		Sex × exposure interaction, *P* value	Boys: multivariable mean difference (95% CI), *P* value	Exposure × cohort interaction, *P* value (boys)	Girls: multivariable model, mean difference (95% CI), *P* value	Exposure × cohort interaction *P* value (girls)
	Univariable model, *P* value	Multivariable model, *P* value					
What are you currently trying to do about your weight? (years included: 2005 and 2015 [n = 18 746])							
Dieting (yes vs no)	0.45 (0.41 to 0.48)	0.37 (0.33 to 0.41)	<.001	0.26 (0.20 to 0.32)	.11	0.49 (0.43 to 0.54)	<.001
Lifetime exercise for weight loss (years included: 1986 and 2015 [n = 18 913])							
Exercise for weight loss (yes vs no)	0.23 (0.20 to 0.27)	0.26 (0.22 to 0.30)	<.001	0.13 (0.08 to 0.19)	.30	0.38 (0.32 to 0.44)	<.001
Lifetime dieting (years included: 1986 and 2015 [n = 18 913])							
Lose weight (vs do nothing)	0.47 (0.43 to 0.51)	0.39 (0.34 to 0.44)	<.001	0.23 (0.17 to 0.29)	.99	0.51 (0.45 to 0.58)	.09
Stay same (vs do nothing)	0.01 (–0.03 to 0.05)	–0.01 (–0.06 to 0.03)	.13	0.03 (–0.02 to 0.08)	.55	–0.04 (–0.11 to 0.02)	.23
Gain weight (vs do nothing)	0.16 (0.08 to 0.23)	0.24 (0.17 to 0.30)	.01	0.18 (0.10 to 0.26)	.10	0.37 (0.22 to 0.51)	.77
Lifetime exercise for weight loss (years included: 1986 and 2015 [n = 18 913])							
Underweight (vs right weight)	0.25 (0.19 to 0.30)	0.27 (0.21 to 0.33)	.89	0.26 (0.19 to 0.34)	^b^	0.29 (0.20 to 0.38)	^c^
Overweight (vs right weight)	0.42 (0.38 to 0.45)	0.38 (0.36 to 0.41)	.01	0.27 (0.20 to 0.33)	^d^	0.44 (0.38 to 0.49)	^e^

^a^ Multivariable models were adjusted for adolescent’s sex, BMI, and ethnicity; maternal age and highest level of education; and paternal social class. We additionally fit an interaction to the multivariable model to test for the presence of sex-specific associations and present sex-stratified models. In these we test for a cohort-by-exposure interaction to test for cohort effects. All analyses based on a sample of participants with at least one outcome available at age 14 years (16 years in 1986) and imputed missing covariate; we additionally used attrition weights to account for attrition at this sweep since recruitment.

^b^ Underweight × Avon Longitudinal Study of Parents and Children (ALSPAC) *P* = .09; underweight × Millennium Cohort Study (MCS) *P* = .21.

^c^ Underweight × ALSPAC *P* = .01; underweight × MCS *P* = .37.

^d^ Overweight × ALSPAC *P* = .21; overweight × MCS *P* = .33.

^e^ Overweight × ALSPAC *P* = .08; overweight × MCS *P* < .001.

**Figure 2.**
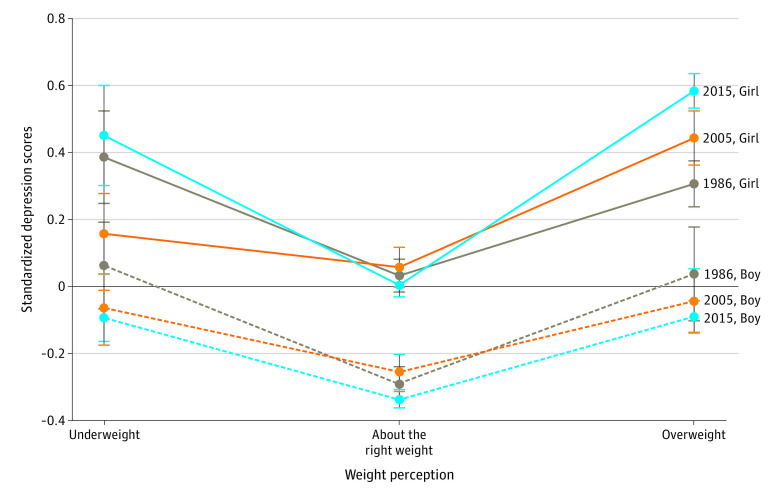
Changes in the Association Between Weight Perception and Depressive Symptoms by Cohort and Sex Parameters derived from multivariate linear regression models in Table 3 with exposure × cohort interaction and sex-stratified analyses presented in eTable 7 in the Supplement.

Adolescents who thought they were underweight and those who thought they were overweight had greater depressive symptoms (mean difference underweight: 0.27 [95% CI, 0.21-0.33]; overweight: 0.38 [95% CI, 0.36-0.41]). There was evidence of a weight perception-by-sex interaction only among adolescents who said they were overweight, with girls who thought they were overweight reporting greater depressive symptoms than boys (mean difference girls: 0.44 [95% CI,0.38-0.49]; boys: 0.27 [95% CI, 0.20-0.33]). There was also evidence that for girls, the magnitude of this association increased in 2015 (mean difference: 0.62 [95% CI, 0.54-0.69]) compared with 1986 (mean difference: 0.32 [95% CI, 0.17-0.30]) and 2005 (mean difference 0.35 [95% CI, 0.24-0.46]) (Table 3, Figure 2, and eTable 7 in the Supplement).

### Sensitivity Analyses

Models with complete cases (eTables 8 and 10 in the Supplement) and with imputed data sets without attrition weights (eTables 9 and 11 in the Supplement) demonstrate results not substantially different from those presented as main analyses. In MCS, prevalence and regression estimates did not vary from those in the main analyses when accounting for stratified sampling (eTables 12, 13, and 14 in the Supplement).

## Discussion

Examining 3 cohorts of adolescents born across 30 years in the UK, our study results suggest key trends in weight change–related behaviors that have occurred in parallel with decades of increasing child obesity. We also investigated the association of weight-change behaviors with depressive symptoms and observed that they were associated with an increased psychological burden in recent years compared with previous decades.

Our results suggest that the prevalence of weight-change behaviors increased in 2015 compared with both 2005 and 1986. Although behaviors aimed at weight loss were more common in girls in all cohorts, their prevalence increased more in boys in recently born cohorts; this was also observed in other countries.^15,16^ In line with US evidence,^30^ in our sample, weight-gain attempts were more common in boys and became increasingly prevalent in this group over 15 years. Recent evidence suggests that over the past couple of decades there has been a shift in media representation of male beauty ideals, with lean muscular bodies increasingly being normalized, which could explain our findings.^31,32,33^ By contrast, pressures on women to be thin have been present for longer in society, with increases in dieting ads documented since the 1960s and 1970s in the US^34,35,36,37^ and becoming more common in the UK in the 1980s and 1990s.^38^

The prevalence of exercise for weight loss, on the other hand, increased from 1986 to 2015 in both boys and girls. Evidence suggests that the proportion of adolescents engaging in vigorous physical activity has remained relatively stable over the past few decades.^39,40^ It is possible that the growing narrative around physical activity as a way to prevent or reduce overweight and obesity—reflected in recent controversial calls to add exercise-equivalent labels on food packaging^41^ might have led adolescents to think of exercise predominantly as a means to lose weight. Although exercise can be effective at reducing body weight,^6^ evidence suggests that the motivation behind exercise, such as wanting to lose weight and feeling guilty if not exercising, are important indicators of negative psychopathology, including depressive and eating disorder symptoms.^42^ Public health campaigns and clinicians should therefore consider the potential negative implications of how messages around physical activity are delivered. These campaigns should not foster feelings of guilt or shame but rather highlight broader positive aspects of exercise, such as improving well-being and strength, learning new skills, and socializing with friends.

It is noteworthy that the trends we observed were not explained by changes in BMI across cohorts and that adolescents increasingly overestimated their weight in more recent cohorts, albeit with different patterns in boys and girls. In 2015 and 2005, boys in the normal weight range became more likely to think they were overweight compared with 1986, whereas girls became more likely to think their weight was about right when underweight in 2015 compared with 1986 and 2005. Greater public health focus on calorie restriction and exercise,^43,44,45,46^ the proliferation of the fitness industry,^47^ and growing societal and media portrayals of lean female and male bodies^48,49,50,51,52^ could have led to adolescents’ increasingly internalizing thin body ideals^53^ and weight stigma, which are known correlates and predictors of restrictive eating behaviors, poor self-esteem, and depression.^54,55^

A recent systematic review on young people’s view on body image and weight in the UK found that children with higher BMIs report appearance-based bullying resulting in social isolation and low mood^56^ and that young people think it is a person’s responsibility to maintain a healthy weight—an idea often reinforced by media^57^—leading to high levels of self-blame for failing to lose weight.^56^ A Finnish study observed similar patterns around blame^19^ that find correspondence in our findings of increasing depressive symptoms associated with the thought of being overweight and weight-loss behaviors in girls over the years. Although in our study it was not possible to disentangle the direction of associations between weight-perception and weight-change behaviors and depressive symptoms, it is important that families, schools, and clinicians are aware of this comorbidity when interacting with adolescents about weight-related concerns.

### Limitations

This study has some limitations. Although BCS and MCS are national cohorts, the ALSPAC cohort is limited to children born in a southwest region of England and might therefore not be representative of other areas of the UK. To account for observed differences in the makeup of the cohorts, we have included a number of sociodemographic and socioeconomic variables in our analyses. Overall, the inclusion of covariates did not affect the results of our analyses, suggesting that between-cohort sampling differences are unlikely to explain the observed cohort trends. All of these cohorts are affected by different degrees of attrition, which could have introduced selection bias in our analyses. To address this, we have imputed missing data for any individual who had at least 1 outcome measurement and used attrition weights in our analyses. The depression measure included in BCS differed from that used in ALSPAC and MCS, which could have resulted in underestimating or overestimating differences between cohorts if adolescents with weight-control behaviors report their depressive symptoms differently on these 2 measures. However, we believe this is unlikely to have occurred, as we observed increased depressive symptoms in 2015 compared with 2005 when measured using the same instrument and did not see changes in boys (but did in girls), which we would have expected if differences were solely due to the instruments used. Future studies should collect data on anxiety, which is common in adolescence and also associated with disordered eating.^58^ The exercise question only focused on exercise for weight loss but not weight gain. Evidence suggests that exercising to increase muscularity is becoming more prevalent, particularly in boys^33^; this is something that studies should consider capturing in the future.

## Conclusions

This study’s findings suggest that the proportion of adolescents who were trying to lose weight at age 14 years has increased over the past 30 years. We acknowledge that there are health concerns associated with obesity; however, the finding that 44% of adolescents aged 14 years were dieting in 2015 is concerning in light of evidence that dieting is generally ineffective for weight loss and is longitudinally associated with weight gain and poor mental health.^7,8,9,10,11,12,13^ Importantly, we found that the association between dieting behaviors and overweight perception and depressive symptoms in girls has increased in magnitude over the past 30 years. Although our study could not directly measure this, it is possible that mounting societal pressures to lose weight could be becoming more detrimental for young people’s mental health and that they could be a contributor to the rising prevalence of adolescent mental health disorders. Early adolescence is a crucial developmental period, when dieting could have a range of negative outcomes, from delayed growth to eating disorders.^59,60^ Reducing the prevalence of restrictive eating behaviors and weight dissatisfaction should be considered an important public health priority in itself, and these behaviors should not only be viewed as problematic when occurring alongside eating disorder diagnoses or in adolescents with low BMI. Public health campaigns around obesity should include prevention of disordered eating behaviors by addressing weight stigma and avoiding the use of body dissatisfaction as a motivator for weight change; advocating for health as opposed to “healthy weight” or “thinness”; promoting family meals; and encouraging adolescents to exercise for health, well-being, and socialization rather than as a means to achieve weight loss.^45,61^

## Notes

Supplement. **eMethods 1.** Cohorts **eMethods 2.** Measures of Depressive Symptoms **eMethods 3.** Body Mass Index **eMethods 4.** Multiple Imputation **eMethods 5.** Changes From Published Protocol **eTable 1.** Summary of Harmonized Measures Across the Three Cohorts **eTable 2.** Sociodemographic Characteristics of the 3 Samples: Sample Based on Participants With Data Available for at Least 1 Outcome Variable (N = 22 503) **eTable 3.** Predictors of Data Missing by Cohort (N = 49 314) **eTable 4.** Prevalence of Weight-Loss Behaviors and Weight Perception Across Cohorts (Sex-Stratified Results are Presented in Table 1) **eTable 5.** Results of Multivariable Analyses in Table 1 Further Adjusted for BMI **eTable 6.** Prevalence of Weight Perception by Cohort, BMI Category, and Child’s Sex Based on Imputed Data set With Attrition Weights (N = 22 503) **eTable 7.** Linear Regression Models Testing the Association Between Lifetime Dieting and Exercising and Attempts to Lose/Gain Weight and Depressive Symptoms Stratified by Sex and Cohort **eTable 8.** Univariable and Multivariable Logistic Regression Models Testing Cohort Effects in the Prevalence of Lifetime Dieting and Exercising for Weight Loss and Interactions With Adolescent’s Sex (Sample of Participants With Complete Cases) **eTable 9.** Univariable and Multivariable Logistic Regression Models Testing Cohort Effects in the Prevalence of Lifetime Dieting and Exercising for Weight Loss and Interactions With Adolescent’s Sex. (Data set Fully Imputed for all Participants) **eTable 10.** Linear Regression Models Testing the Association Between Lifetime Dieting and Exercising; and Attempts to Lose/Gain Weight and Depressive Symptoms Stratified by Sex and Cohort (Complete Cases) **eTable 11.** Linear Regression Models Testing the Association Between Lifetime Dieting and Exercising; and Attempts to Lose/Gain Weight and Depressive Symptoms Stratified by Sex and Cohort (Fully Imputed Data set) **eTable 12.** Prevalence of Weight-Change Behaviors in the Millennium Cohort Study (2015) Calculated Accounting for the Stratified Sampling Design (Corresponding Main Analysis in Table 1) **eTable 13.** Prevalence of Weight Perception by Actual BMI Category in the Millennium Cohort Study (2015) Calculated Accounting for the Stratified Sampling Design (Corresponding Main Analysis in eTable6) **eTable 14.** Linear Regression Models Testing the Association Between Lifetime Dieting and Exercising; and Attempts to Lose/Gain Weight and Depressive Symptoms Stratified by Sex and Cohort, Comparing MCS Stratified Estimates Obtained With and Without Accounting for the Stratified Sampling Design. (Corresponding Main Analysis in eTable7)
eReferences
