# Supplementary material for: Changes in the Prevalence and Correlates of Weight-Control Behaviors and Weight Perception in Adolescents in the UK, 1986-2015
Source: JAMA Pediatr. 2020 Nov 16;175(3):267–75. doi: 10.1001/jamapediatrics.2020.4746 (PMC7670392; doi:10.1001/jamapediatrics.2020.4746)
Supplement: Supplement. — eMethods 1. Cohorts eMethods 2. Measures of Depressive Symptoms eMethods 3. Body Mass Index eMethods 4. Multiple Imputation eMethods 5. Changes From Published Protocol eTable 1. Summary of Harmonized Measures Across the Three Cohorts eTable 2. Sociodemographic Characteristics of the 3 Samples: Sample Based on Participants With Data Available for at Least 1 Outcome Variable (N = 22 503) eTable 3. Predictors of Data Missing by Cohort (N = 49 314) eTable 4. Prevalence of Weight-Loss Behaviors and Weight Perception Across Cohorts (Sex-Stratified Results are Presented in Table 1) eTable 5. Results of Multivariable Analyses in Table 1 Further Adjusted for BMI eTable 6. Prevalence of Weight Perception by Cohort, BMI Category, and Child’s Sex Based on Imputed Data set With Attrition Weights (N = 22 503) eTable 7. Linear Regression Models Testing the Association Between Lifetime Dieting and Exercising and Attempts to Lose/Gain Weight and Depressive Symptoms Stratified by Sex and Cohort eTable 8. Univariable and Multivariable Logistic Regression Models Testing Cohort Effects in the Prevalence of Lifetime Dieting and Exercising for Weight Loss and Interactions With Adolescent’s Sex (Sample of Participants With Complete Cases) eTable 9. Univariable and Multivariable Logistic Regression Models Testing Cohort Effects in the Prevalence of Lifetime Dieting and Exercising for Weight Loss and Interactions With Adolescent’s Sex. (Data set Fully Imputed for all Participants) eTable 10. Linear Regression Models Testing the Association Between Lifetime Dieting and Exercising; and Attempts to Lose/Gain Weight and Depressive Symptoms Stratified by Sex and Cohort (Complete Cases) eTable 11. Linear Regression Models Testing the Association Between Lifetime Dieting and Exercising; and Attempts to Lose/Gain Weight and Depressive Symptoms Stratified by Sex and Cohort (Fully Imputed Data set) eTable 12. Prevalence of Weight-Change Behaviors in the Millennium Cohort Study (2015) Calculated Accoun [file jamapediatr-e204746-s001.pdf]

Supplementary Online Content

Solmi F, Sharpe H, Gage SH, Maddock J, Lewis G, Patalay P. Changes in the prevalence and correlates of weight-control behaviors and weight perception in adolescents in the UK, 1986-2015. *JAMA Pediatr*. Published online November 16, 2020. doi:10.1001/jamapediatrics.2020.4746

eMethods 1. Cohorts

eMethods 2. Measures of Depressive Symptoms

eMethods 3. Body Mass Index

eMethods 4. Multiple Imputation

eMethods 5. Changes From Published Protocol

eTable 1. Summary of Harmonized Measures Across the Three Cohorts

eTable 2. Sociodemographic Characteristics of the 3 Samples: Sample Based on Participants With Data Available for at Least 1 Outcome Variable (N = 22 503)

eTable 3. Predictors of Data Missing by Cohort (N = 49 314)

eTable 4. Prevalence of Weight-Loss Behaviors and Weight Perception Across Cohorts (Sex-Stratified Results are Presented in Table 1)

eTable 5. Results of Multivariable Analyses in Table 1 Further Adjusted for BMI

eTable 6. Prevalence of Weight-Perception by Cohort, BMI Category, and Child's Sex Based on Imputed Dataset With Attrition Weights (N = 22 503)

eTable 7. Linear Regression Models Testing the Association Between Lifetime Dieting and Exercising and Attempts to Lose/Gain Weight and Depressive Symptoms Stratified by Sex and Cohort

eTable 8. Univariable and Multivariable Logistic Regression Models Testing Cohort Effects in the Prevalence of Lifetime Dieting and Exercising for Weight-Loss and Interactions With Adolescent's Sex (Sample of Participants With Complete Cases)

eTable 9. Univariable and Multivariable Logistic Regression Models Testing Cohort Effects in the Prevalence of Lifetime Dieting and Exercising for Weight-Loss and Interactions With Adolescent's Sex. (Dataset Fully Imputed for all Participants)

eTable 10. Linear Regression Models Testing the Association Between Lifetime Dieting and Exercising; and Attempts to Lose/Gain Weight and Depressive Symptoms Stratified by Sex and Cohort (Complete Cases)

© 2020 Solmi F et al. *JAMA Pediatrics*.

eTable 11. Linear Regression Models Testing the Association Between Lifetime Dieting and Exercising; and Attempts to Lose/Gain Weight and Depressive Symptoms Stratified by Sex and Cohort (Fully Imputed Dataset)

eTable 12. Prevalence of Weight-Change Behaviors in the Millennium Cohort Study (2015) Calculated Accounting for the Stratified Sampling Design (Corresponding Main Analysis in Table 1)

eTable 13. Prevalence of Weight-Perception by Actual BMI Category in the Millennium Cohort Study (2015) Calculated Accounting for the Stratified Sampling Design (Corresponding Main Analysis in eTable6)

eTable 14. Linear Regression Models Testing the Association Between Lifetime Dieting and Exercising; and Attempts to Lose/Gain Weight and Depressive Symptoms Stratified by Sex and Cohort, Comparing MCS Stratified Estimates Obtained With and Without Accounting for the Stratified Sampling Design. (Corresponding Main Analysis in eTable7)

eReferences

This supplementary material has been provided by the authors to give readers additional information about their work.

© 2020 Solmi F et al. *JAMA Pediatrics*.

eMethods 1. Cohorts

The 1970 British Cohort Study (BCS) is birth cohort study which aimed to recruit 17,287 children born between 5 and 11 April, 1970, in England, Scotland, Wales, and Northern Ireland. The initial study sample consisted of 16,567 (95.8%) children and their mothers, who have been followed up since via postal questionnaires, medical examination and record linkage. Ethics approval for BCS70 was obtained for all sweeps after the year 2000. Prior sweeps did not receive formal external approval (but did receive internal approval), in line with the regulations of the time.[1]

The Avon Longitudinal Study of Parents and Children (ALSPAC), is a birth cohort that recruited 14,541 pregnancies with an expected delivery date falling between 1st April 1991 and 31st December 1992 in the region of Avon. These pregnancies resulted in 14,062 live births, with 13,988 children alive at one year of age. The ALSPAC Law and Ethics committee and the Local Research Ethics committees gave ethical approval for the study. The study website (www.bristol.ac.uk/alspac) provides more information on the sample and contains details of all the data that is available through a fully searchable data dictionary available at: http://www.bris.ac.uk/alspac/researchers/data-access/data-dictionary/.

The Millennium Cohort Study (MCS) is a longitudinal birth cohort of 18,818 children born between 1st September 2000 and 11th January 2002, who were living in the United Kingdom (UK) at 9 months, and were eligible for receiving child benefits. The cohort includes children living in non-household situations and children who were not born in the UK, but lived in the UK at recruitment. The study used a stratified clustered framework to ensure disadvantaged and ethnic minority groups were adequately represented.[14] The Multi-Centre Research Ethics Committee (MREC) gave ethics approval for MCS.

Participants gave written consent to take part in these studies.

© 2020 Solmi F et al. *JAMA Pediatrics*.

eMethods 2. Measures of Depressive Symptoms

The Short Moods and Feeling Questionnaire[2] contains 13 statements which the participant could respond in one of three ways (0 = “not true”, 1 =”sometimes”, 2=”true”) whose total score ranges from 0 to 26, with greater scores indicating greater depressive symptoms. The 9 questions from the Malaise inventory are scored on a three-point Likert scale (0 = rarely/never, 1 = some of the time, 2 = most of the time), resulting in a total score ranging from 0 to 27, with higher scores indicating greater symptoms.[3]

eMethods 3. Body Mass Index

To minimize data missingness, in BCS and ALSPAC we supplemented objective measures of BMI (obtained by fieldworkers in BCS and in clinic assessments in ALSPAC) with self-reported measurements when objective measures were not available (28% of participants in BCS, 11% in ALSPAC). The correlation between the two was good (r=0.69 BCS and r=0.89 ALSPAC). In MCS, fieldworkers objectively measured BMI at study visit with no self-reported measures available. In all samples, we created age- and sex-standardized BMI categories indicating underweight, normal weight, and overweight BMI using the International Obesity Task Force cut-offs. [4–6]

eMethods 4. Multiple Imputation

We imputed missing outcome and covariate data for participants with at least one outcome variable available using multiple imputation by chained equations and imputing 50 datasets.

In our imputation models, we included all variables used in the analyses, plus an indicator of maternal marital status, as these variables could be harmonized across datasets. We imputed data separately for each cohort and merged them after imputation for analyses. To account for participant attrition from baseline assessments in each dataset, we created attrition weights as the inverse of the probability of having taken part in the sweep of interest. We used indicators of child’s sex and ethnicity, paternal social class, and maternal marital status, age, and highest education level to create attrition weights. To do this, we first imputed missing baseline data using single imputation as the proportion of participants in our sample with missing data was minimal (2% BCS, 9% ALSPAC, 22% MCS*), so that all children in our sample would have an attrition weight. We ran all of our analyses in imputed datasets using attrition weights. We additionally ran a number of sensitivity analyses using complete cases and imputed data without weights to check the consistency of our results.

© 2020 Solmi F et al. *JAMA Pediatrics*.

*all MCS missing data were on the social class variable. There was no missingness on maternal age, marital status, child’s ethnicity and sex.

eMethods 5. Changes From Published Protocol

We made minimal changes to the analysis plan detailed in our protocol these were:

1. We additionally included attrition weights in the analyses to account for attrition within cohorts
2. We ran a model further adjusting for BMI for the the analyses looking at changes in prevalence of current weight intentions.

© 2020 Solmi F et al. *JAMA Pediatrics*.

eTable 1. Summary of Harmonized Measures Across the 3 Cohorts

**BCS70**		**ALSPAC**	**MCS**	**Harmonized measure**
Do you think you are: under/over or right weight?<br>1. Underweight<br>2. Overweight<br>3. About the right weight		How do you describe your weight:<br>4. Very underweight<br>5. Slightly underweight<br>6. About the right weight<br>7. Slightly overweight<br>8. Very overweight	Which of these do you think you are<br>1. Underweight<br>2. About the right weight<br>3. Slightly overweight<br>4. Very overweight	Adolescent perceive themselves as:<br>1. Underweight<br>2. About the right weight<br>3. Overweight
N/A		Which of the following are you trying to do about your weight?<br>1. I am not trying to do anything about my weight<br>2. Stay the same<br>3. Gain weight<br>4. Lose weight	What are you trying to do about your weight?<br>1. Lose weight<br>2. Gain weight<br>3. Stay the same weight<br>4. I am not trying to do anything about my weight	Adolescent is trying to:<br>1. Not trying to do anything about their weight<br>2. Stay the same weight<br>3. Lose weight<br>4. Gain weight
Depressive symptoms (Malaise inventory)		Depressive symptoms (SMFQ)	Depressive symptoms (SMFQ)	Depressive symptoms (standardized SMFQ and Malaise inventory scores)
BMI categories using IOTF cut offs<br>1. grade 3 thinness<br>2. grade 2 thinness<br>3. grade 1 thinness<br>4. normal weight<br>5. overweight<br>6. obese		BMI categories using IOTF cut offs<br>1. grade 3 thinness<br>2. grade 2 thinness<br>3. grade 1 thinness<br>4. normal weight<br>5. overweight<br>6. obese	BMI using IOTF cut offs<br>1. grade 3 thinness<br>2. grade 2 thinness<br>3. grade 1 thinness<br>4. normal weight<br>5. overweight<br>6. obese	BMI using IOTF cut-offs<br>1. Underweight<br>2. Normal weight<br>3. Overweight<br>4. Obese
Have you ever tried to lose weight?<br>1. no<br>2. yes<br>(if yes)<br>have you dieted to lose weight?<br>1. no<br>2. yes<br>have you exercised to lose weight?<br>1. no<br>2. yes		-N/A	Have you ever dieted to lose weight?<br>1. no<br>2. yes<br><br>Have you ever exercised to lose weight?<br>1. no<br>2. yes	Have you ever dieted to lose weight?<br>1. no<br>2. yes<br><br>Have you ever exercised to lose weight?<br>3. no<br>4. yes

© 2020 Solmi F et al. *JAMA Pediatrics*.

eTable 2. Sociodemographic Characteristics of the Three Samples, Sample Based on Participants With Data Available for at Least 1 Outcome Variable (N = 22503**)**

	**Total**	**1986**	**2005**	**2015**
	**N (%)**	**n (%)**	**n (%)**	**n (%)**
**Total**	22,503	5,878	5,832	10,793
**Sex**				
Male	10,442 (46.4%)	2,514 (42.8%)	2,600 (44.6%)	5,328 (49.4%)
Female	12,061 (53.6%)	3,364 (57.2%)	3,232 (55.4%)	5,465 (50.6%)
**Ethnicity***				
White British	19,942 (89.9%)	4,664 (95.5%)	5,356 (96.1%)	8,922 (82.7%)
BAME	2,239 (10.1%)	148(2.5%)	220 (4.0%)	1,871 (17.3%)
**Maternal Education**				
Compulsory	14,229 (63.9%)	4,585 (78.5%)	3,110 (55.0%)	6,534 (60.7%)
Non-compulsory	8,026 (36.1%)	1,255 (21.5%)	2,547 (45.0%)	4,224 (39.3%)
**Paternal social class**				
Manual	8,826 (44.7%)	3,666 (62.5%)	1,972 (36.4%)	3,188 (37.7%)
Non-manual	10,917 (55.3%)	2,204 (37.5%)	3,448 (63.6%)	5,265 (62.3%)
**Adolescents' BMI** [a]				
Underweight	1,700 (8.4%)	570 (11.0%)	428 (8.7%)	702 (6.8%)
Normal weight	14,558 (71.1%)	4,028 (77.5%)	3,604 (73.3%)	6,926 (66.9%)
Overweight	3,171 (15.5%)	494 (9.5%)	709 (14.4%)	1,968 (19.0%)
Obese	1,034 (5.0%)	103 (2.0%)	175 (3.6%)	756 (7.3%)
	**Mean (SD)**	**Mean (SD)**	**Mean (SD)**	**Mean (SD)**
Adolescent's age	14.8 (0.3)	16.5 (0.5)	14.0 (0.4)	14.3 (0.3)
Maternal Age	28.6 (5.6)	26.1 (5.3)	29.2 (4.6)	29.8 (5.8)

List of abbreviations: ALSPAC = Avon Longitudinal Study of Parents and Children; BCS1907 = British Cohort Study 1970; BMI = Body Mass Index; MCS = Millennium Cohort Study.
[a] cut-offs derived from age and sex standardized values

There were key differences across cohort, which broadly reflect changing population demographics. Across all cohorts the majority of the sample was female (53.6%) and of white ethnicity (89.9%), but compared to BCS (2.5%) and ALSPAC (4.0%), in MCS the number of the proportion of BAME adolescents was higher (17.3%). Compared to ALSPAC (45.0%) and MCS (39.3%), in BCS fewer adolescents had a mother who had completed non-compulsory education (21.5%). Similarly, in BCS the minority of children had a father working in a non-manual occupation (37.5%), but this increased both in ALSPAC (63.6%) and MCS (62.3%). Maternal age at birth of study child and BMI increased throughout cohorts. Compared to BCS, the proportion of adolescents with an overweight (9.5%) or obese (2.0%) BMI was higher in ALSPAC (overweight: 14.4%, obese: 3.6%) and MCS (overweight: 19.0%, obese: 7.3%).

© 2020 Solmi F et al. *JAMA Pediatrics*.

*Ethnicity was defined as follow in the different cohorts:

- BCS70: Contains a binary variable which is provided coded as: white/ethnic minority. Further details are not provided.
- ALSPAC: Contains a binary variable which is provided coded as: white/non-white. Further details are not provided.
- MCS: Ethnicity is defined using Office for National Statistics categories which we recoded as: white /ethnic minority to harmonize this variable across cohorts. Original categories were: white, mixed, Indian, Pakistani, Bangladeshi, Other Asian, black Caribbean, black African, other black, Chinese, and other ethnic group.

© 2020 Solmi F et al. *JAMA Pediatrics*.

eTable 3. Predictors of Data Missing by Cohort (N = 49 314)

	**Missing BCS 11,099 (65.4%)**	**Missing ALSPAC 7,956 (57.7%)**	**Missing MCS 7,756 (41.8%)**
	**Odds Ratio (95%CI)**	**Odds Ratio (95%CI)**	**Odds Ratio (95%CI)**
**Sex**			
Male	Reference	Reference	Reference
Female	0.57 (0.54, 0.61)	0.61 (0.57, 0.66)	0.82 (0.64, 0.73)
**Ethnicity**			
White British	Reference	Reference	Reference
Ethnic minorities	1.63 (1.34, 1.98)	1.55 (1.31, 1.84)	1.01 (0.94, 1.09)
**Maternal Education**			
Compulsory	Reference	Reference	Reference
Non-compulsory	0.66 (0.62, 0.72)	0.45 (0.42, 0.49)	0.53 (0.49, 0.56)
**Paternal social class**			
Manual	Reference	Reference	Reference
Non-manual	0.66 (0.62, 0.71)	0.56 (0.52, 0.61)	0.68 (0.64, 0.73)
**Maternal Age**	0.99 (0.98, 0.99)	0.92 (0.91, 0.92)	0.96 (0.95, 0.96)

© 2020 Solmi F et al. *JAMA Pediatrics*.

eTable 4. Prevalence of Weight-Loss Behaviors and Weight Perception Across Cohorts (Sex-Stratified Results are Presented in Table 1)

	**BCS (1986) n=5,889**	**ALSPAC (2005) n=5,832**	**MCS (2015) n=10,796**
	**% (95% CI)**	**% (95% CI)**	**% (95% CI)**
**Lifetime dieting** [a]			
No	62.3 (60.9 – 63.7)	NA	55.4 (54.4 – 56.4)
Yes	37.7 (36.3 – 39.1)	NA	44.4 (43.1 – 45.6)
**Lifetime exercising for weight loss** [a]			
No	93.2 (92.4 – 93.9)	NA	39.5 (38.6 – 40.5)
Yes	6.8 (6.0- 7.6)	NA	60.4 (59.5 – 61.4)
**What are you doing about your weight:** [b]			
Not doing anything	NA	38.0 (36.6 – 39.3)	23.6 (22.6 – 24.7)
Lose weight	NA	29.8 (28.6 – 31.1)	42.2 (40.9 – 43.4)
Stay the same	NA	27.0 (25.8 – 28.3)	25.6 (24.6 – 26.7)
Gain weight	NA	5.2 (4.6 – 5.9)	8.5 (7.8 – 9.2)
**Do you think you are:** [c]			
Underweight	12.4 (11.5 – 13.4)	14.4 (13.4 – 15.4)	7.2 (6.7– 7.7)
About the right weight	65.4 (64.2 – 66.7)	58.6 (57.3 – 60.0)	59.5 (58.4 – 60.3)
Overweight	22.2 (21.1 – 23.2)	27.0 (25.7 – 28.2)	33.4 (32.5 – 34.3)

[a] sample size: n = 16,671
[b] sample size: n = 16,625
[c] sample size: n = 22,503

© 2020 Solmi F et al. *JAMA Pediatrics*.

eTable 5. Results of Multivariable Analyses in Table 1 Further Adjusted for BMI

	**Multivariable Model**<br>**males**	**Multivariable Model**<br>**females**
	**OR (95%CI)**<br>**p-value**	**OR (95%CI)**<br>**p-value**
**What are you trying to do about your weight:**		
	**RRR (95%CI)**<br>**p-value**	**RRR (95%CI)**<br>**p-value**
Nothing	Reference outcome	Reference outcome
Lose weight<br>*MCS (2015) vs ALSPAC (2005)*	2.42 (2.05, 2.86), p<0.0001	1.40 (1.22, 1.60), p<0.0001
Stay the same<br>*MCS (2015) vs ALSPAC (2005)*	1.86 (1.61, 2.13), p<0.0001	1.14 (0.99, 1.31), p=0.07
Gain weight<br>*MCS (2015) vs ALSPAC (2005)*	2.50 (1.99, 3.13), p<0.0001	1.98 (1.42, 2.76), p=0.0001

© 2020 Solmi F et al. *JAMA Pediatrics*.

eTable 6. Prevalence of Weight-Perception by Cohort, BMI Category, and Child's Sex Based on Imputed Dataset With Attrition Weights (N = 22 503)

**Actual Body Mass Index**									
**Males: Do you think you are?**	**Underweight BMI (n= 805) % (95%CI)**			**Normal Weight (n=7,565) % (95%CI)**			**Overweigh/Obese BMI (n=2,072) % (95%CI)**		
	**1986**	**2005**	**2015**	**1986**	**2005**	**2015**	**1986**	**2005**	**2015**
Underweight	49.5% (42.7%-56.3%)	57.6% (49.7%-65.4%)	44.7% (39.6%-49.7%)	13.2% (11.6%-14.8%)	16.9% (15.2%-18.7%)	9.4% (8.5%-10.5%)	2.0% (0.2%-3.8%)	1.4% (0.3%-2.5%)	0.6% (0.1%-1.0%)
Normal weight	48.8% (42.1%-55.6%)	39.6% (31.8%-47.4%)	54.0% (49.3%-59.4%)	79.5% (77.6%-81.4%)	71.5% (69.4%-73.6%)	79.0% (77.6%-80.3%)	37.6% (31.4%-43.7%)	24.1% (19.9%-28.4%)	26.3% (23.9%-28.7%)
Overweight	1.6% (0.1%-3.3%)	2.8% (0.1%-5.4%)	1.0% (0.1%-1.9%)	7.3% (6.1%-8.5%)	11.5% (10.0%-13.0%)	11.5% (10.5%-12.6%)	60.3% (54.1%-66.6%)	74.5% (70.2%-78.8%)	73.1% (70.7%-75.5%)
**Actual Body Mass Index**									
**Females: Do you think you are?**	**Underweight BMI (n= 928) % (95%CI)**			**Normal Weight (n=8,449) % (95%CI)**			**Overweigh/Obese BMI (n=2,684) % (95%CI)**		
	**1986**	**2005**	**2015**	**1986**	**2005**	**2015**	**1986**	**2005**	**2015**
Underweight	44.3% (38.9%-49.8%)	51.6% (45.2%-58.0%)	35.4% (30.2%-40.8%)	5.7% (4.8%-6.7%)	10.3% (9.0%-11.6%)	3.9% (3.2%-4.6%)	1.6% (0.3%-2.9%)	1.6% (0.5%-2.7%)	0.1% (0.0%-0.2%)
Normal weight	51.7% (46.2%-57.1%)	42.3% (36.0%-48.6%)	60.8% (55.4%-66.3%)	65.5% (63.5%-67.4%)	69.3% (67.3%-71.2%)	72.2% (70.7%-73.7%)	16.4% (12.6%-20.1%)	19.2% (16.1%-22.3%)	18.6% (16.7%-20.6%)
Overweight	4.0% (1.9%-6.1%)	6.1% (2.7%-9.4%)	3.4% (1.6%-5.8%)	28.8% (27.0%-30.6%)	20.4% (18.7%-22.1%)	23.8% (22.4%-25.3%)	82.0% (78.1%-85.9%)	79.2% (76.0%-82.4%)	81.3% (79.4%-83.2%)

© 2020 Solmi F et al. *JAMA Pediatrics*.

eTable 7. Linear Regression Models Testing the Association Between Lifetime Dieting and Exercising and Attempts to Lose/Gain Weight and Depressive Symptoms Stratified by Sex and Cohort

	**Depressive symptoms in Males**			**Depressive symptoms in Females**		
	1986: Multivariable Model Mean difference (95% CI)	2005: Multivariable Model Mean difference (95% CI)	2015: Multivariable Model Mean difference (95% CI)	1986: Multivariable Model Mean difference (95% CI)	2005: Multivariable Model Mean difference (95% CI)	2015: Multivariable Model Mean difference (95% CI)
**Lifetime dieting**						
Yes (vs no)	0.22 (0.09, 0.35)	NA	0.29 (0.23, 0.34)	0.21 (0.12, 0.29)	NA	0.72 (0.66. 0.78)
**Lifetime exercising for weight loss**						
Yes (vs no)	0.05 (-0.17, 0.27)	NA	0.13 (0.08, 0.18)	0.06 (-0.08, 0.20)	NA	0.46 (0.39, 0.53)
**Are you currently trying to:**						
Lose weight (vs do nothing)	NA	0.23 (0.11, 0.35)	0.22 (0.15, 0.27)	NA	0.43 (0.31, 0.54)	0.58 (0.50, 0.66)
Stay same (vs do nothing)	NA	0.03 (-0.05, 0.14)	0.01 (-0.04, 0.06)	NA	0.01 (-0.11, 0.11)	-0.08 (-0.16, -0.01)
Gain weight (vs do nothing)	NA	0.27 (0.08. 0.45)	0.14 (0.06, 0.21)	NA	0.39 (0.12, 0.66)	0.35 (0.19, 0.51)
**Do you think you are:**						
Underweight (vs right weight)	0.33 (0.17, 0.48)	0.19 (0.07, 0.31)	0.26 (0.17, 0.35)	0.30 (0.15, 0.46)	0.13 (-0.01, 0.28)	0.42 (0.27, 0.59)
Overweight (vs right weight)	0.37 (0.21, 0.53)	0.21 (0.08, 0.33)	0.23 (0.17, 0.30)	0.32 (0.22, 0.42)	0.35 (0.24, 0.46)	0.62 (0.54, 0.69)

[a] adjusted for: adolescent's sex, age, BMI, and ethnicity; maternal age and highest level of education; and paternal social class. [b] n= 16,685 [c] n= 16,904 [d]n =22,503

© 2020 Solmi F et al. *JAMA Pediatrics*.

eTable 8. Univariable and Multivariable Logistic Regression Models Testing Cohort Effects in the Prevalence of Lifetime Dieting and Exercising for Weight-Loss and Interactions With Adolescent's Sex (Sample of Participants With Complete Cases)

	Univariable model	Multivariable[a] Model	Sex*cohort interaction p-value	Multivariable Model In males	Multivariable Model females
	OR (95%CI)	OR (95%CI)	OR (95%CI)	OR (95%CI)	OR (95%CI)
**Lifetime dieting for weight loss[b]**					
1986	Reference	Reference		Reference	Reference
2015	1.12 (1.03, 1.20)	1.46 (1.16, 1.85)	p<0.001	1.79 (1.24, 2.60)	1.29 (0.94, 1.76)
**Lifetime exercising for weight loss[b]**					
1986	Reference	Reference		Reference	Reference
2015	19.62 (17.39, 22.12)	30.37 (22.79, 40.49)	p=0.27	-	-
	RRR (95%CI)	RRR (95%CI)	Sex*cohort interaction	RRR (95%CI)	RRR (95%CI)
**What are you currently trying to do about your weight? (Comparing MCS to ALSPAC, reference cohort)[c]**					
Doing nothing	Reference outcome	Reference outcome		Reference outcome	Reference outcome
Lose weight (2015 vs 2005)	2.16 (1.97, 2.36)	2.15 (1.94, 2.38)	p<0.001	2.75 (2.36, 3.23)	1.74 (1.52, 1.99)
Stay Same (2015 vs 2005)	1.51 (1.37, 1.65)	1.52 (1.37, 1.70)	p=0.09	1.92 (1.66, 2.23)	1.19 (1.02, 1.38)
Gain weight (2015 vs 2005)	2.67 (2.27, 3.14)	1.99 (1.66, 2.39)	p=0.05	2.29 (1.84, 2.85)	1.68 (1.21, 2.32)
**Do you think you are: (Comparing MCS and ALSPAC to BCS, reference cohort)[d]**					
Underweight (2005 vs 1986)	1.32 (1.17, 1.49)	1.60 (1.06, 2.42)	p=0.89	1.99 (1.15, 3.45)	1.21 (0.64, 2.29)
Underweight (2015 vs 1986)	0.64 (0.57, 0.73)	0.73 (0.50, 1.06)	p=0.32	0.96 (0.59, 1.56)	0.51 (0.28, 0.91)
About the right weight	Reference outcome	Reference outcome	Reference outcome	Reference outcome	Reference outcome
Overweight (2005 vs 1986)	1.21 (1.10, 1.33)	1.60 (1.17, 2.19)	p<0.001	2.77 (1.63, 4.72)	1.17 (0.79, 1.75)
Overweight (2015 vs 1986)	1.43 (1.32, 1.55)	1.36 (1.04, 1.80)	p<0.001	2.39 (1.51, 3.79)	1.00 (1.11, 1.68)

[b] n=12,995; [c] n=13,142 ; [d] n=17,384

© 2020 Solmi F et al. *JAMA Pediatrics*.

eTable 9. Univariable and Multivariable Logistic Regression Models Testing Cohort Effects in the Prevalence of Lifetime Dieting and Exercising for Weight-Loss and Interactions With Adolescent's Sex. (Dataset Fully Imputed for all Participants)

	Univariable model	Multivariable[a] Model	Sex*cohort interaction p-value	Multivariable Model In males	Multivariable Model females
	OR (95%CI)	OR (95%CI)	OR (95%CI)	OR (95%CI)	OR (95%CI)
**Lifetime dieting for weight loss[b]**					
1986	Reference	Reference		Reference	Reference
2015	1.33 (1.24, 1.43)	1.55 (1.23, 1.95)	p<0.001	1.79 (1.24, 2.59)	1.23 (0.91, 1.66)
**Lifetime exercising for weight loss[b]**					
1986	Reference	Reference		Reference	Reference
2015	20.92 (18.42, 23.76)	26.65 (20.07, 35.40)	p=0.27	-	-
	RRR (95%CI)	RRR (95%CI)	Sex*cohort interaction	RRR (95%CI)	RRR (95%CI)
**What are you currently trying to do about your weight? (Comparing MCS to ALSPAC, reference cohort)[c]**					
Doing nothing	Reference outcome	Reference outcome		Reference outcome	Reference outcome
Lose weight (2015 vs 2005)	2.28 (2.10, 2.48)	2.18 (1.98, 2.39)	p<0.001	2.75 (2.38, 3.19)	1.70 (1.50, 1.92)
Stay Same (2015 vs 2005)	1.51 (1.38, 1.65)	1.52 (1.38, 1.68)	p<0.001	1.88 (1.63, 2.16)	1.15 (1.00, 1.32)
Gain weight (2015 vs 2005)	2.62 (2.25, 3.05)	1.99 (1.68, 2.35)	P=0.01	2.32 (1.87, 2.85)	1.53 (1.14, 2.07)
**Do you think you are: (Comparing MCS and ALSPAC to BCS, reference cohort)[d]**					
Underweight (2005 vs 1986)	1.30 (1.15, 1.46)	1.49 (1.00, 2.21)	p=0.73	1.89 (1.12, 3.18)	1.00 (0.54, 1.82)
Underweight (2015 vs 1986)	0.64 (0.57, 0.72)	0.72 (0.51, 1.03)	p=0.22	0.97 (0.61, 1.53)	0.43 (0.25, 0.76)
About the right weight	Reference outcome	Reference Outcome	Reference outcome	Reference outcome	Reference outcome
Overweight (2005 vs 1986)	1.36 (1.24, 1.49)	1.64 (1.22, 2.19)	p<0.001	3.06 (1.82, 5.15)	1.01 (0.71, 1.44)
Overweight (2015 vs 1986)	1.66 (1.54, 1.79)	1.47 (1.14, 1.90)	p<0.001	2.59 (1.66, 4.06)	0.95 (0.69, 1.30)

[b] n=35,526; [c] n=32,337 ; [d] n=49,314

© 2020 Solmi F et al. *JAMA Pediatrics*.

eTable 10. Linear Regression Models Testing the Association Between Lifetime Dieting and Exercising; and Attempts to Lose/Gain Weight and Depressive Symptoms Stratified by Sex and Cohort (Complete Cases)

	**Depressive symptoms in Males**			**Depressive symptoms in Females**		
	1986: Multivariable Model Mean difference (95% CI)	2005: Multivariable Model Mean difference (95% CI)	2015: Multivariable Model Mean difference (95% CI)	1986: Multivariable Model Mean difference (95% CI)	2005: Multivariable Model Mean difference (95% CI)	2015: Multivariable Model Mean difference (95% CI)
**Lifetime dieting** (n=12,995)						
Yes (vs no)	0.24 (0.10, 0.38)	NA	0.29 (0.23, 0.34)	0.21 (0.11, 0.30)	NA	0.73 (0.66. 0.80)
**Lifetime exercising for weight loss** (n=12,995)						
Yes (vs no)	0.06 (-0.18, 0.30)	NA	0.15 (0.09, 0.20)	0.05 (-0.10, 0.20)	NA	0.49 (0.41, 0.56)
**Are you currently trying to: (**n=13,142)						
Lose weight (vs do nothing)	NA	0.17 (0.06, 0.28)	0.22 (0.15, 0.28)	NA	0.46 (0.33, 0.58)	0.60 (0.50, 0.69)
Stay same (vs do nothing)	NA	0.04 (-0.05, 0.13)	0.01 (-0.07, 0.06)	NA	0.01 (-0.11, 0.12)	-0.06 (-0.15, 0.04)
Gain weight (vs do nothing)	NA	0.21 (0.06. 0.37)	0.10 (0.01, 0.18)	NA	0.43 (0.13, 0.72)	0.42 (0.23, 0.60)
**Do you think you are:** (n=17,384)						
Underweight (vs right weight)	0.29 (0.15, 0.43)	0.23 (0.12, 0.34)	0.24 (0.16, 0.33)	0.31 (0.15, 0.46)	0.11 (-0.03, 0.27)	0.39 (0.22, 0.56)
Overweight (vs right weight)	0.31 (0.15, 0.47)	0.17 (0.06, 0.28)	0.25 (0.18, 0.31)	0.35 (0.24, 0.45)	0.35 (0.24, 0.47)	0.62 (0.53, 0.70)

© 2020 Solmi F et al. *JAMA Pediatrics*.

eTable 11. Linear Regression Models Testing the Association Between Lifetime Dieting and Exercising; and Attempts to Lose/Gain Weight and Depressive Symptoms Stratified by Sex and Cohort (Fully Imputed Dataset)

	**Depressive symptoms in Males**			**Depressive symptoms in Females**		
	1986: Multivariable Model Mean difference (95% CI)	2005: Multivariable Model Mean difference (95% CI)	2015: Multivariable Model Mean difference (95% CI)	1986: Multivariable Model Mean difference (95% CI)	2005: Multivariable Model Mean difference (95% CI)	2015: Multivariable Model Mean difference (95% CI)
**Lifetime dieting** (n=35,526)						
Yes (vs no)	0.21 (0.12, 0.31)	NA	0.39 (0.24, 0.44)	0.20 (0.12, 0.28)	NA	0.65 (0.59, 0.70)
**Lifetime exercising for weight loss** (n=35,526)						
Yes (vs no)	0.06 (-0.09, 0.23)	NA	0.20 (0.15, 0.25)	0.05 (-0.08, 0.19)	NA	0.42 (0.36, 0.48)
**Are you currently trying to:** (n=32,337)						
Lose weight (vs do nothing)	NA	0.34 (0.24, 0.45)	0.32 (0.25, 0.38)	NA	0.42 (0.32, 0.52)	0.52 (0.45, 0.59)
Stay same (vs do nothing)	NA	0.03 (-0.05, 0.11)	-0.01 (-0.07, 0.05)	NA	0.02 (-0.08, 0.11)	-0.06 (-0.14, 0.02)
Gain weight (vs do nothing)	NA	0.29 (0.09, 0.49)	0.19 (0.11, 0.27)	NA	0.37 (0.15, 0.57)	0.32 (0.18, 0.46)
**Do you think you are:** (n=49,314)						
Underweight (vs right weight)	0.33 (0.23, 0.43)	0.18 (0.08, 0.28)	0.29 (0.21, 0.38)	0.31 (0.19, 0.42)	0.13 (0.01, 0.26)	0.39 (0.26, 0.51)
Overweight (vs right weight)	0.34 (0.22, 0.45)	0.26 (0.15, 0.38)	0.34 (0.28, 0.40)	0.32 (0.22, 0.41)	0.33 (0.24, 0.42)	0.56 (0.49, 0.62)

© 2020 Solmi F et al. *JAMA Pediatrics*.

eTable 12. Prevalence of Weight-Change Behaviors in the Millennium Cohort Study (2015) Calculated Accounting for the Stratified Sampling Design (Corresponding Main Analysis in Table 1)

	**MCS (2015)**		
	**All children % (95% CI)**	**Boys %(95%CI)**	**Girls % (95%CI)**
**Lifetime dieting**	44.6% (43.7% – 45.5%)	34.6% (33.3% - 35.8%)	55.1% (53.8% - 56.5%)
**Lifetime exercising**	60.5% (59.5% - 61.4%)	54.9% (53.5% - 56.3%)	66.3% (65.0% - 67.6%)
**What are you trying to do about weight**			
Lose weight	42.2% (41.2% - 43.1%)	31.8% (30.6%- 33.1%)	53.0% (51.7%-54.3%)
Stay the same	25.5% (24.6% - 26.4%)	28.3% (27.1%-29.6%)	22.5% (21.4%-23.6%)
Gain weight	8.5% (8.0% - 9.0%)	12.7% (11.8%- 13.6%)	4.1% (3.6%-4.6%)
**Do you think you are?**			
Underweight	7.2% (6.7% -7.7%)	-[a]	-[a]
About the right weight	59.4% (58.3% - 60.4%)	-[a]	-[a]
Overweight	33.4% (32.5% - 34.3%)	-[a]	-[a]

[a] presented stratified by BMI category in eTable13

© 2020 Solmi F et al. *JAMA Pediatrics*.

eTable 13. Prevalence of Weight-Perception by Actual BMI Category in the Millennium Cohort Study (2015) Calculated Accounting for the Stratified Sampling Design (Corresponding Main Analysis in eTable6)

	**Actual BMI**		
**Do you think you are?**	**Underweight BMI % (95%CI)**	**Normal weight BMI % (95%CI)**	**Overweigh/Obese BMI % (95%CI)**
**Males**			
Underweight	44.7% (39.5% - 49.8%)	9.4% (8.5%-10.5%)	0.6% (0.2%-1.0%)
About the right weight	54.3% (49.1% - 59.5%)	79.0% (77.7%-80.3%)	26.3% (24.0%-28.5%)
Overweight	1.0% (0.01% - 1.9%)	11.5% (10.5%-12.6%)	73.1% (70.9%-75.3%)
**Females**			
Underweight	35.5% (30.3%-40.7%)	3.9% (3.2%-4.6%)	0.1% (0.0%-0.2%)
About the right weight	60.8% (55.4%-66.2%)	72.2% (70.7%-73.7%)	18.6% (16.7%-20.6%)
Overweight	3.7% (1.7%-5.7%)	23.9% (22.4%-25.3%)	81.3% (79.3%-83.3%)

© 2020 Solmi F et al. *JAMA Pediatrics*.

eTable 14. Linear Regression Models Testing the Association Between Lifetime Dieting and Exercising; and Attempts to Lose/Gain Weight and Depressive Symptoms Stratified by Sex and Cohort, Comparing MCS Stratified Estimates Obtained With and Without Accounting for the Stratified Sampling Design. (Corresponding Main Analysis in eTable7)

Depressive symptoms				
	Boys		Girls	
	2015: Multivariable Model Mean difference (95% CI)<br><br>[without accounting for sampling strata]	2015: Multivariable Model Mean difference (95% CI)<br><br>[accounting for sampling strata]	2015: Multivariable Model Mean difference (95% CI)<br><br>[without accounting for sampling strata]	2015: Multivariable Model Mean difference (95% CI)<br><br>[accounting for sampling strata]
Lifetime dieting				
Yes (vs no)	0.29 (0.23, 0.34)	0.29 (0.23, 0.34)	0.72 (0.66. 0.78)	0.76 (0.66, 0.78)
Yes (vs no)	0.13 (0.08, 0.18)	0.13 (0.09, 0.18)	0.46 (0.39, 0.53)	0.46 (0.40, 0.52)
Lose weight (vs do nothing)	0.22 (0.15, 0.27)	0.22 (0.16, 0.28)	0.58 (0.50, 0.66)	0.58 (0.50, 0.66)
Stay same (vs do nothing)	0.01 (-0.04, 0.06)	0.01 (-0.04, 0.06)	-0.08 (-0.16, -0.01)	-0.08 (-0.16, -0.01)
Gain weight (vs do nothing)	0.14 (0.06, 0.21)	0.13 (0.06, 0.21)	0.35 (0.19, 0.51)	0.35 (0.19, 0.51)
Underweight (vs right weight)	0.26 (0.17, 0.35)	0.26 (0.18, 0.35)	0.42 (0.27, 0.59)	0.43 (0.26, 0.61)
Overweight (vs right weight)	0.23 (0.17, 0.30)	0.24 (0.17, 0.30)	0.62 (0.54, 0.69)	0.62 (0.54, 0.69)

© 2020 Solmi F et al. *JAMA Pediatrics*.

eReferences

1. Shepherd P. *1970 British Cohort Study Ethical Review and Consent*.; 2012.

2. Angold A, Costello EJ, Messer SC, Pickles A, Winder F, Silver D. Development of a short questionnaire for use in epidemiological studies of depression in children and adolescents. *Int J Methods Psychiatr Res*. 1995;5:237-249.

3. Rutter M. *Education, Health and Behaviour*. Harlow: Longman; 1970.

4. Vidmar SI, Cole TJ, Pan H. Standardizing anthropometric measures in children and adolescents with functions for egen: Update. *Stata J*. 2013;13(2):366-378.

5. Cole TJ, Flegal KM, Nicholls D, Jackson AA. Body mass index cut offs to define thinness in children and adolescents: international survey. *BMJ*. 2007;335(7612).

6. Cole TJ, Bellizzi MC, Flegal KM, Dietz WH. Establishing a standard definition for child overweight and obesity worldwide: international survey. *BMJ*. 2000;320(7244).

© 2020 Solmi F et al. *JAMA Pediatrics*.
